# Crystal orientation-dependent fatigue characteristics in micrometer-sized single-crystal silicon

**DOI:** 10.1038/micronano.2016.27

**Published:** 2016-07-18

**Authors:** Tsuyoshi Ikehara, Toshiyuki Tsuchiya

**Affiliations:** 1 National Institute of Advanced Industrial Science and Technology (AIST), 1-2 Namiki, Tsukuba, Ibaraki 305-8564, Japan; 2 Department of Micro Engineering, Graduate School of Engineering, Kyoto University, Kyotodaigaku-Katsura C3, Nishikyo-ku, Kyoto 615-8540, Japan

**Keywords:** fatigue, fracture, lifetime, MEMS, reliability, resonator, single-crystal silicon

## Abstract

Repetitive bending fatigue tests were performed using five types of single-crystal silicon specimens with different crystal orientations fabricated from {100} and {110} wafers. Fatigue lifetimes in a wide range between 10^0^ and 10^10^ were obtained using fan-shaped resonator test devices. Fracture surface observation via scanning electron microscope (SEM) revealed that the {111} plane was the primary fracture plane. The crack propagation exponent *n* was estimated to be 27, which was independent of the crystal orientation and dopant concentration; however, it was dependent on the surface conditions of the etched sidewall. The fatigue strengths relative to the deflection angle were orientation dependent, and the ratios of the factors obtained ranged from 0.86 to 1.25. The strength factors were compared with those obtained from finite element method stress analyses. The calculated stress distributions showed strong orientation dependence, which was well-explained by the elastic anisotropy. The comparison of the strength factors suggested that the first principal stress was a good criterion for fatigue fracture. We include comparisons with specimens tested in our previous report and address the tensile strength, initial crack length, volume effect, and effects of surface roughness such as scallops.

## Introduction

Historically, single crystals have been rarely used as a structural material; however, single-crystal silicon (SCS) emerged in the 1960s as a primary material for micromechanical structures^1^, which are now called microelectromechanical systems (MEMS). Micro movable structures are fabricated from SCS or polycrystalline silicon (PS) mainly using micro-lithography and etching techniques. Such small parts are subjected to repetitive stresses applied by various external forces including electrostatic force, inertial force, thermal stress and hydrofluidic force. In the case of vibrational operations, repetition frequencies are typically on the order of kilohertz and sometimes reach gigahertz levels for radio frequency applications. These devices are operated not only in an inert environment, such as in vacuum or dry nitrogen gas, but also, for example, in humid air or water for chemical sensor applications.

Mechanical reliability assessment of silicon structures has been performed using statistical treatments^2^ based on its brittle nature at room temperature (RT). The Weibull statistical distribution is usually utilized for designing fracture reliabilities^3^. However, typical micro-fabrication technologies provide rather poor relative structural accuracies and surface conditions compared with bulk structures owing to the limited number of available fabrication techniques in the machining and surface treatment processes. Therefore, MEMS typically require much larger tolerance in the strength design than conventional bulk structures do.

Anisotropy is another mechanical issue unique to single-crystal materials. The anisotropic properties in SCS have been well-known for MEMS designers in chemical aspects such as anisotropic etching technology and also in electrical aspects including the piezoresistive effect. However, MEMS designers have been forced to be aware of mechanical anisotropy since the 1990s as mechanical design and evaluation techniques have been refined. Anisotropic materials generally have two different aspects in their mechanical properties: elastic anisotropy and fracture anisotropy. Elastic anisotropy is understood within the classical continuum mechanism and can now be easily introduced into the design by employing the anisotropic stiffness constants *c*_*ij*_ in finite element method (FEM) simulation or analytical calculation instead of isotropic elastic constants such as Young’s modulus^4–6^. Elastic anisotropy sometimes appears significantly in non-uniaxial systems, including curved or cranked beams and diaphragms.

However, the fracture anisotropy of a crystal is governed by atomic bonding behavior that cannot be analyzed with continuum mechanics^7^. As yet, we have little empirical strength data on the SCS, unlike familiar anisotropic materials, woods, fiber reinforced plastics, and other composite materials. We have no idea how to include specific crystal planes, such as the well-known {111} or {110} cleavage planes, in the fracture design of microstructures. In other words, it is still unclear regarding which stress component should be responsible for fracture: principal stress, normal stress or shear stress on a unique crystal plane. Through orientation-dependent micro-tensile tests on SCS, the {111} plane has been observed as a primary cleavage surface at RT^8–10^. Normal stress on the {111} plane has been proposed as a good fracture criterion from the statistical analysis of micro-tensile tests^9^. Contrarily, by nanometer-sized fracture testing, shear stress on {111} has also been noted as a criterion in a wide temperature region, including RT^11^. Furthermore, principal stress or von Mises stress has been practically employed as a criterion for fracture analyses, which tacitly assumes the absence of fracture anisotropy.

Delayed fracture owing to the fatigue of SCS and PS microstructures has been gradually more fully understood in recent years. Fatigue fracture is a significant issue for the reliability assessment of MEMS subjected to repetitive stresses. The mechanisms of fatigue have not yet been unveiled; however, important experimental findings have exhibited the following salient points:

- Fatigue rate is strongly dependent on the environment, particularly on humidity^12–18^. Fatigue is largely suppressed in vacuum or inert environments. These results imply the significance of the surface oxidation effect in subcritical fatigue progress.
- The slopes of stress or strain to lifetime (*S*–*N*) plots have been strongly dependent on specimens, test methods, and environments^11,16,17,19–22^, although this is an important parameter to express the fatigue crack growth. The reason for this variation is still unknown.
- Stress ratio dependence has been confirmed^23,24^. The compressive phase in the stress cycle was found to be significant, as was the tensile phase that opens the fatigue crack. Debris effects have been discussed for that reason.
- Both positive and negative results have been reported on the frequency dependence and the existence of static fatigue^14,21,25^. The fatigue lifetime has usually been treated as the number of loading cycles rather than the loading time.
- Microscopic material behavior around the crack tip at the nanometer scale, which cannot be directly observed by lifetime methods, has been investigated by various material evaluation techniques. The behavior is still not clearly understood. Ductile deformation along the {111} plane has been reported in sub-micrometer specimens at lower temperatures than the brittle-to-ductile transition temperature or even at RT^26–31^. However, size-independent fracture toughness has also been reported^32^ below 100 nm, which might imply that brittleness is maintained.

We have performed fatigue lifetime measurements on micrometer-sized SCS bending specimens using a fan-shaped rotational resonator, which is widely utilized for material characterization. Such test devices^33,34^ have the advantage that they exclude uncertainties resulting from clamping difficulties for micro-sized specimens. We have carefully prepared precisely fabricated specimens^35^ and a test system with amplitude-controlled oscillation^36^ to obtain the *S*–*N* characteristics in a wide lifetime range. The measured lifetimes have been discussed assuming the propagation velocity of the crack of length *a* as

(1)dadt=C[KKc]n

where *K*, *K*_c_, *n*, and *C* are the stress intensity factor, the fracture toughness, the crack growth exponent, and a constant, respectively. The solution^17^ under continuous sinusoidal loading gives the relationship between the maximum stress $\sigma_{f}^{\left(c\right)}$ and the lifetime cycles $N_{f}^{\left(c\right)}$

(2)Nf(c)−1=a0C′22−n[σf(c)σ0]−2(1−[σf(c)σ0]2−n)

where the initial crack length *a*_0_ and the tensile fracture stress *σ*_0_ without fatigue have the relationship

(3)Kc=βσ0πa0

where *β* is a correction factor relating to crack geometry and *C*' is a constant. By analyzing the *S*–*N* characteristics using these equations, we have thus far confirmed humidity dependence^17,35^, crystal orientation dependence^37^, and the surface roughness effect^38^. Such delicate dependences were difficult to resolve in a microscale fatigue test using external actuation^39^.

In this report, we extend the variety of crystal orientations of the specimen to examine the detailed effects of anisotropy including cleavage planes such as {111}. In the previous specimens on a {100} wafer plane, the relationship between the stress state and the {111} planes could not be altered drastically by laterally moving deformation. We added three other crystal configurations using a {110} wafer to provide more varied stress conditions to change the stress conditions on the {111} plane. The orientation-dependent *S*–*N* relationships of five types of specimens were measured. We compared the orientation dependence with the stress distributions calculated using FEM analyses and discussed the methodology for the design of fatigue fracture.

## Materials and methods

### Fatigue test device

**Figure 1:**
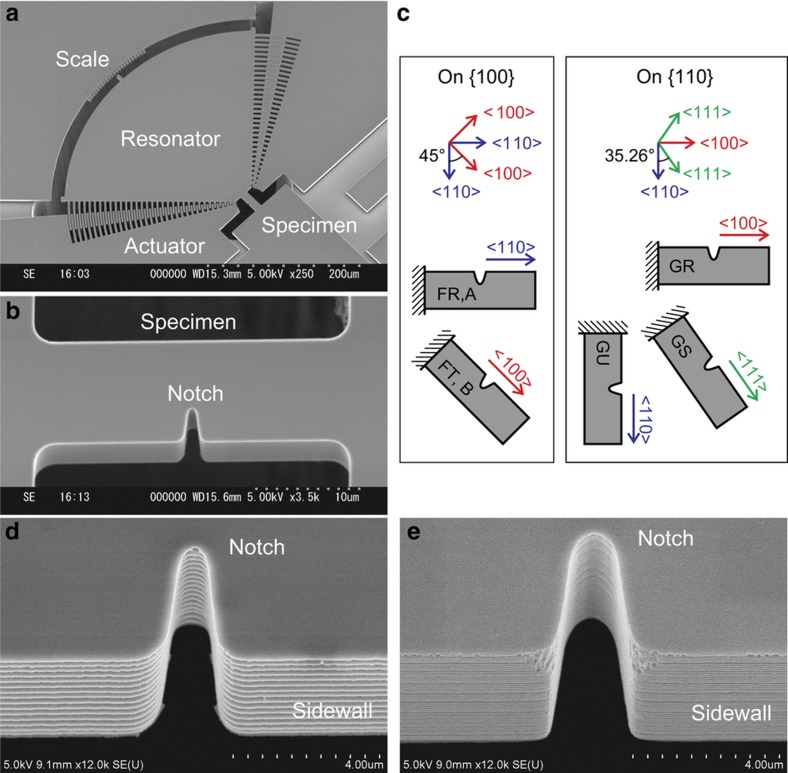
Fatigue test specimens. Bird’s-eye view SEM pictures are shown for (**a**) fan-shaped resonator and (**b**) notched specimen. Configuration of specimens on the photomask and corresponding crystal orientations on {100} and {110} silicon wafers are illustrated in (**c**). SEM pictures of the sidewall around the notch are shown in (**d**) currently and (**e**) previously fabricated specimens.

The fatigue test device consists of a notched rectangular specimen (length 30 μm, width 10 μm, height 5 μm (Figure 1b)) and a fan-shaped resonator (radius 250 μm) with two comb electrodes (Figure 1a). We fabricated five types of specimens with different crystal orientation configurations, as shown in Figure 1c, from two types of SCS wafers. The specimens were designed to lie along representative orientations in a cubic crystal, <100>, <110>, and <111> in the wafer crystal planes, whereas the structure and its dimensions are completely identical. On the {100} wafer, type FR (along <110>) and FT (along <100>) specimens were fabricated from one sheet of {100} bonded silicon-on-insulator (SOI) wafer. On the {110} wafer, type GU (along <110>), GR (along <100>), and GS (along <111>) specimens were fabricated from one sheet of {110} bonded SOI wafer. These five types along with two types A and B that were used in previous reports^17,35,37^ are summarized in Table 1. We indicate the specimen types by these short specimen names in this paper to distinguish the different types. Collective names, types Fx and Gx, are also used to include all types from {100} wafers (types FR and FT) and {110} wafers (types GU, GR, and GS), respectively. Miller indices {hkl} and <hkl> are used without distinguishing equivalent directions.

### Fabrication process and material

Two types of bonded-type SOI wafers were used as the specimen materials, where the SCS device layer (thickness 5 μm) was grown using the Czochralski method with boron dopant. The materials of the two wafers could not be completely identical (Table 1), and the surface finishing might differ because these wafers were obtained from different wafer suppliers owing to availability. The devices were processed within the same fabrication lot employing in-house facilities with 1.0 μm resolution technology. All rotated specimen patterns were included in a single photomask and etched simultaneously by deep reactive ion etching (DRIE). The handle layer below the device was then removed by backside DRIE, and the buried oxide layer was removed by dry HF etching. Finally, the wafers were divided into chips by a laser dicer and die-bonded on an open ceramic package with conductive epoxy resin. The lithography and etching conditions were maintained within the lot to fabricate specimens with the same qualities as much as possible.

However, the process conditions had to be different from the previous fabrication for types A and B, for which an outsourcing MEMS foundry was employed^35^. Compared with the previous lot by scanning electron microscope (SEM) observation (Figures 1d and e), although an identical photomask design was employed, the resulting notch shapes (depth 3.4 μm, tip radius 0.3 μm) of the new specimens were slightly shrunken from the previous ones (depth 4.0 μm, tip radius 0.5 μm) owing to the inferior lithography resolution. In addition, the sidewall scallops using our finest Bosch-type recipe (period 300 nm, height 50 nm) were somewhat rougher than those in the previous specimen (period 100 nm, height 20 nm), which probably employed a high-frequency alternating recipe. The effects of the notch shape and its sidewall roughness are discussed later. The change in the resonator gain owing to gap narrowing was another effect of the lithography process alteration. The resonator gain change was compensated by the adjustment of the electric circuit gain. We recalibrated the signal-to-deflection angle factor by observing the deflection scale under oscillation^35^.

### Fatigue test

The electrostatically driven resonators were self-oscillated at the resonant frequencies using an amplitude-controlled feedback-oscillation circuit in which the vibration amplitudes were controlled by an automatic gain controller (AGC). The resonant frequencies are type dependent, as shown in Table 1, owing to the anisotropic flexural rigidity. Two test procedures, constant amplitude (CA) and ramping amplitude (RA), were performed. The CA test was a simple fatigue-life test for relatively long life conditions in which the loading amplitude levels were set to give fatigue lifetime from 10^5^ to 10^11^ cycles. The RA test employed oscillation with linearly raised amplitude at a constant rate, in which the CA-equivalent lifetimes $N_{f}^{\left(c\right)}$ were estimated from the observed lifetime $N_{f}^{\left(r\right)}$ using the relationship $N_{f}^{\left(c\right)}=N_{f}^{\left(r\right)}/(n+1)$. This is derived from the crack extension law, Equation (1), under ramped sinusoidal loading^17^. By changing the ramping rate, CA-equivalent low-cycle lifetimes could be estimated for the region from 10^0^ to 10^5^ cycles. The stress ratio was fixed at *R*=−1 for both tests owing to the large quality factor of the resonator of ~400 in air. The tests were performed in an environment-controlled chamber at a temperature of 23.0 °C and humidity of 50% RH, where the stabilities were maintained within ±0.1 °C and ±1% RH and the accuracies were within ±0.5 °C and ±3% RH. Specimen failure was detected by monitoring the electric deflection signal from the comb electrode capacitor. This fatigue test was performed under the IEC62047-12 standard^40^. See previous reports^17,36^ for details on the measurement procedures and the test system.

After the fatigue tests, all specimens were inspected under field-emission SEM (Hitachi S-4800H, Tokyo, Japan) to determine whether the fracture originated from an unexpected surface defect. Four tests were performed per test level as a minimum estimation of data scattering under the limited numbers of specimens obtained from a single wafer. In total, 116 tests were conducted with 29 conditions.

## Results and discussion

### Fracture surface observation

**Figure 2:**
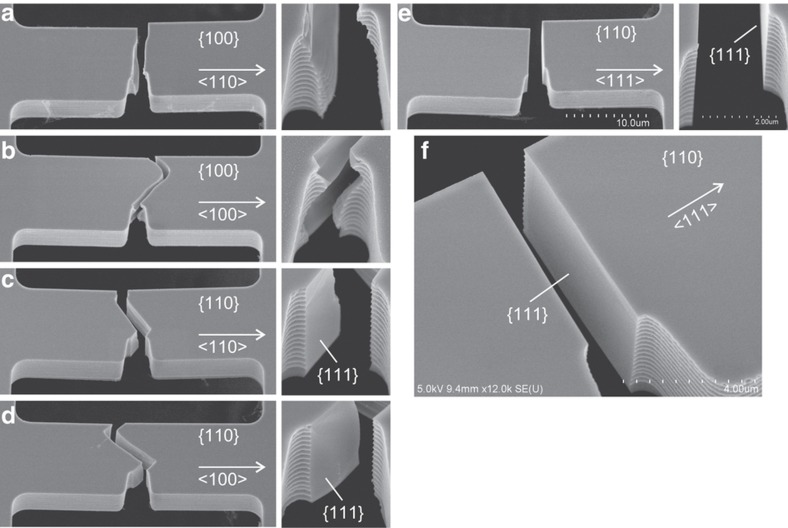
SEM pictures of fatigue-fractured specimens for the five different types: (**a**) FR, (**b**) FT, (**c**) GU, (**d**) GR, and (**e**) GS. High-magnification images around the notch tip are shown at the right. The {111} cleavage surface in type GS is shown in (**f**).

Figures 2a–e show SEM pictures of the specimens after fatigue fracture. All specimens fractured around the notch tip; however, the behavior of the fracture surface development was strongly dependent on the specimen types. First, in the specimens on the {110} plane (Figures 2c–e), distinct {111} planes appeared on the fracture surfaces. The {110} wafer plane includes two <111> directions at the angle of 70.53° as shown in Figure 1c. Therefore, stress in the {110} plane usually provides high normal stress to either of the two vertical {111} planes, that is, high-stress intensity factor *K*_I_ (opening mode) on the {111} plane. In the GU and GR types, the fracture surface propagated alternately along two {111} planes. In particular, in type GS along the <111> direction, a single {111} cleavage mirror was always formed perpendicularly as shown in Figures 2e and f. In contrast, specimens FR and FT on the {100} plane did not exhibit clear crystal planes as shown in Figures 2a and b. Against the {100} wafer plane, all <111> axes inclined by 35.26°. Therefore, it is difficult to maintain stable {111} crack surfaces by a plane stress on the {100} plane. The crack seemed to propagate in the <110> direction by a top view observation. However, by detailed SEM observation as shown in Figures 2a and b, the vertical {110} planes were rarely found, which suggested that the cracks were likely to propagate obliquely, perhaps toward the {111} plane, and the intersection between the {111} and {100} planes produced the {110}-like crack lines observed in the top view. From this observation, the {111} plane was considered as the primary fracture surface in our specimens at RT. This result is consistent with the results of tensile tests^8–10^ and general fracture behavior in brittle semiconductors with a diamond structure^7^.

### Fatigue test

We obtained fatigue lifetimes of 116 specimens for the five types as shown in Table 1. The numbers of test levels were five to seven per type, which were determined as follows:

- For the CA tests, the test levels were set such that the lifetimes of the four specimens were within the measurable range, 2 s to 6.0×10^5^ s (7 days). There were no run-out data.
- For the rapid RA tests using the excessive-gain oscillation method, two test levels were performed with CA-equivalent lifetimes of ~0.15 and 1.5 ms.
- For the amplitude-controlled RA test, 0–2 test levels were performed with the CA-equivalent lifetimes of ~0.07 and 0.7 s.

**Figure 3:**
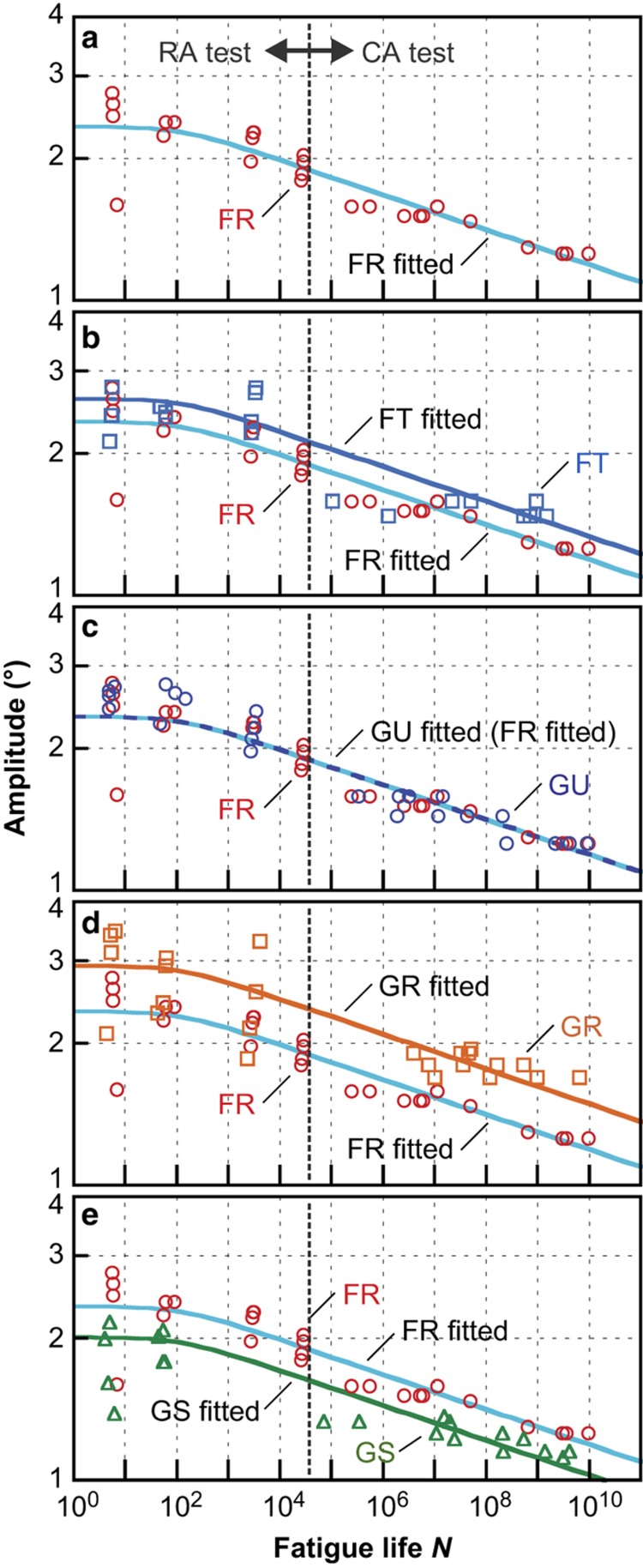
*S*–*N* plots of the fatigue test results and fitted curves for types (**a**) FR, (**b**) FT, (**c**) GU, (**d**) GR, and (**e**) GS. The data and fitted line for type FR are shown in each figure for comparison. The vertical dotted line indicates the boundary of two test procedures.

The numbers of amplitude-controlled RA test levels were type dependent because a higher transient stability was required to control the precisely ramping amplitudes. Delicate changes in the resonant frequencies and quality factors caused small mismatches between the AGC tuning and resonator characteristics, depending on the types. See a previous report^17^ for the detailed methods of the RA tests. Figures 3a–e show the *S*–*N* plots of the fatigue test results for the five types, in log–log scales. The deflection angle amplitude (center to peak) was chosen as the vertical axis for the quantity proportional to the peak stress. The fatigue lifetime on the horizontal axis expresses the number of cycles to failure. The CA-equivalent lifetimes are indicated for the RA test results. The boundary between the CA and RA methods lay at ~5×10^4^ cycles. A general tendency with the linear negative slope was observed.

We discuss the results for the type FR first, in which the maximum test levels were obtained. Figure 3a shows the *S*–*N* plot for the type FR and a fitted line using Equation (2), with the applied stresses substituted for the deflection angles. A clear, linear, negative slope tendency in the log–log scale was observed and could be well-fitted by a straight line with a fatigue crack growth exponent *n*=27 at high cycles. The saturated deflection angle in the low-cycle region *θ*_0_=2.3° was obtained as the averaged value of short lifetime data.

Figures 3b–e show the *S*–*N* plots of the types FT, GU, GR, and GS, respectively, with that of the type FR for comparison. Obvious changes in the curve slope were not found among the five types. Therefore, we assumed that the line shape (slope and saturation) was unchanged among these five types and that only the strength factors (vertical shift) were different. The fitted lines in Figures 3b–e were obtained based on this assumption. The observed strength factors against the type FR are summarized in Table 2. The five types had different fatigue strengths relative to the deflection angle in the order of types GR, FT, FR, GU, and GS. The strengths of types FR and GU were almost comparable.

### Discussion on crack growth exponent *n*

By comparing the results of the five types, clear evidence was obtained that the fatigue crack exponent *n* is independent of the crystal orientation, which implies that the same crack growth mechanism, perhaps on the {111} crystal plane, was mainly involved in fatigue phenomena in SCS. Moreover, it was surprising that the values of *n* were unchanged, although the wafer materials (doping levels and suppliers) were different between types Fx and Gx. The crack growth was considered to be insensitive to the impurity concentration, at least in the case of the boron dopant in the range of 10^15^–10^19^ cm^−3^. These facts are practically significant for simplifying the reliability design of MEMS devices. An *n* value obtained from an *S*–*N* observation can be applicable for other crystal configurations if the structure and the stress-loading conditions are unchanged.

In contrast, the value *n*=27 was much larger than the value *n*=19 obtained for our previous specimens (types A and B) in the same environment (23 °C, 50% RH)^17,37^, although the materials and device dimensions were almost the same. This was confirmed to be a non-environmental effect, although *n* has been found to be humidity dependent. We carefully checked that a constant environment was successfully maintained during the series of our tests by inspecting the performance of the environment-controlled chamber. As a result, the change in *n* might have been caused by the surface conditions owing to the fabrication processes. Because the fabrications were performed in different foundries between two lots (types A, B and types Fx, Gx), there might be differences in, for example, the DRIE machine, its recipe, photoresist removal process, or sacrificial etching process, which can cause differences in the sidewall roughness morphology, surface damage, and surface oxidation thickness. Our results may exhibit strong surface condition dependences on the crack growth exponent *n* in SCS. Further controlled experiments are required to determine the dominant factor.

### Stress analysis using FEM

**Figure 4:**
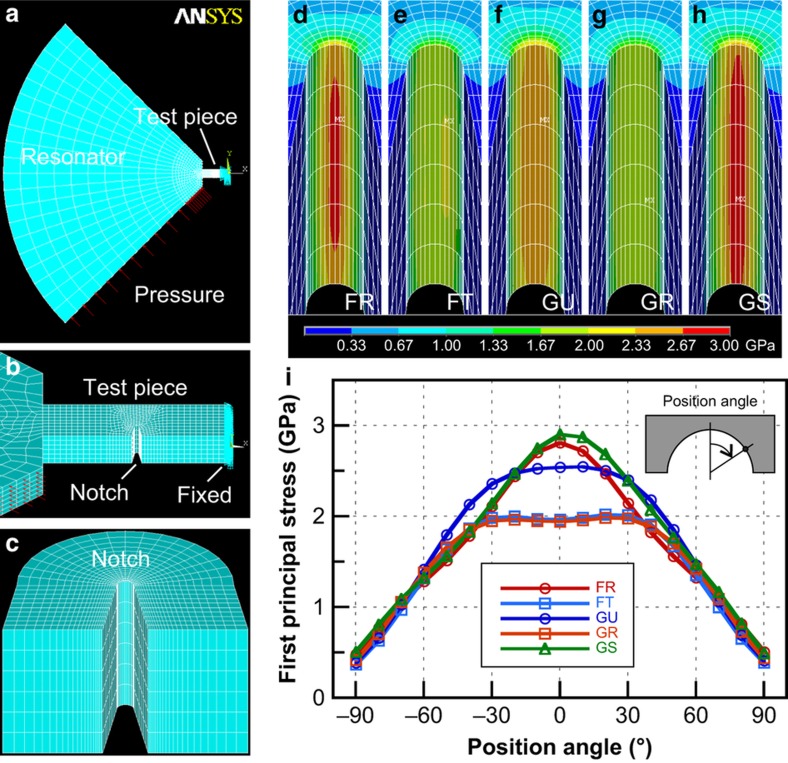
Finite element stress simulation. Finite element models under static deflection are shown in (**a**) and (**b**) for coarse model including the whole resonator. The submodel covering only the vicinity of the notch tip is shown in (**c**). Contour plots of the calculated first principal stress distribution around the notch tip are shown for types (**d**) FR, (**e**) FT, (**f**) GU, (**g**) GR, and (**h**) GS under 1° deflection with the common legend indicated at the bottom. Profiles of the first principal stress distribution along the notch tip are shown in (**i**) where the position is indicated by angles as shown in the inset.

To discuss the fatigue fracture stresses from the measured strength factors relative to the deflection angle, the relationship between the stress and the deflection angle should be estimated. We simulated the stress states under a fixed deflection employing the commercial FEM software program ANSYS 12.0, which allows a proper anisotropic elasticity with three-dimensional 6×6 stiffness tensor as material properties. We used two-dimensional calculations for devices on the {100} plane^4^; however, three-dimensional calculations were introduced in this report to represent the lower structural symmetry in some cases of the {110} plane and to include the thickness effect correctly^41^. The three-dimensional, eight-node element solid185 was used with elastic constants^42^ *c*_11_=167.40 GPa, *c*_12_=65.23 GPa, and *c*_44_=79.57 GPa. A two-step submodeling method under static loading was employed to evaluate the locally concentrated stress distribution around the notch tip in the specimen. The coarse and submodels are shown in Figures 4a–c with element meshing conditions. Crystal orientations were specified as the rotation of the element coordinate system to reproduce the configurations for the five types. The static deflection angle of 1° was induced by a type-dependent pressure on the sidewall at the electrostatic actuator. The nodal displacements of the coarse model were transferred to the submodel as constraints, and the elastic simulations around the notch were finally performed.

Contour plots of the calculated first principal stress distribution on the notch surfaces are shown in Figures 4d–h for the five types with the same legend. The stress distributions were strongly dependent on the types. The stresses became generally higher when the specimens were oriented along the directions of larger flexural rigidity (<111> or <110>). Moreover, a complex tendency was observed: the stress was concentrated at the notch center with high peak values for types FR and GS, whereas it broadened in the notch with relatively low values for types FT and GR. The distributions were more obvious when the profiles at the center plane were plotted along the notch curves as shown in Figure 4i. Types FR and GS exhibited similar curves with high-stress peaks at the notch center. Contrarily, types FT and GR exhibited evenly broad stress distributions with low-peak values at the offset positions from the notch center. Type GU exhibited an intermediate stress distribution. An elasticity-like coefficient *k* (in Pa deg^−1^) was introduced to represent the relationship between the peak stress *σ*_peak_ and the deflection angle *θ* as

(4)σpeak=kθ

The numerically obtained coefficients *k* are summarized in Table 3.

These stress distributions correspond to the fracture origins observed using SEM as shown in Figures 2c–e. The positions of the fracture origins of type Gx on the {110} plane could be easily specified owing to the vertical {111} fracture surfaces, but this was difficult in the case of type Fx. Types GU and GR often exhibited fracture origins with an offset from the notch center, as shown in Figures 2c and d. Type GS typically fractured around the notch center (Figure 2e). These observations are consistent with the stress distribution.

Such orientation dependences are understood by the elastic anisotropy^5,43,44^, given the specimen configurations in Figure 1c. The first principal stress always lies along the surface on the opened smooth surface of the continuum solid. When a tensile plane stress is applied on a vertical surface, the first principal stress tangentially orients along the surface curve. The local stress value increases in proportion to Young’s modulus along the surface curve, even under the same strain^4^. Compared with the crystal axis orientation (Figure 1c), in types FR and GS, the orientations along the surface at the notch center (<110> and <111>) achieved a maximum Young’s modulus on the corresponding wafer planes ({100} and {110}), respectively. Therefore, the stress was concentrated at the notch center. However, in types FT and GR, the orientation along the surface at the notch center (<100>) coincided with the minimum Young’s modulus, and at both sides at 45° and 55° there are directions of the maximum Young’s modulus. Such elastic anisotropy yielded widely distributed stresses along the circular notched structure. Type GU was a special case, in which three orientations (<110> and two <111>) with large Young’s moduli were concentrated within a narrow angle of ±35°. This configuration provides a widely and intermediately concentrated stress distribution.

The foregoing discussion relates to the first principal stress, which was an appropriate criterion to treat the fracture and fatigue of brittle materials with linear, isotropic fracture mechanics. If materials have a special crystal plane for fracture such as a cleavage plane, the normal stress to open such a plane can be a criterion^7^. We further estimated the maximum values of normal stress and additionally for shear stresses on four {111} planes from the stress tensor, and these are summarized in Table 3.

### Comparison with experimental fatigue strengths

If fatigue is governed by a single stress criterion, the strength factors relative to the deflection angle *θ* should be proportional to *k*^−1^ from Equation (4). The estimated strength factors *k*_FR_/*k* against type FR are summarized in Table 2. The coefficient *k* depends on the specimen geometry, applied load, crystal orientation, and selected stress component. Because the specimen geometry and the loading are identical for our specimens, the orientation dependence of *k* directly reflects the stress component that is valid for fatigue fracture criterion. By comparing the orientation dependence of the strength factors with the experimental one, the first principal stress exhibited better matching with the fatigue strength, especially on the order of five types within the three possibilities.

The fact that the principal stress was appropriate as a fatigue criterion implied that the fracture characteristic was nearly isotropic and approximately independent of crystal orientation. This seemed inconsistent with the existence of the primary cleavage {111} plane in SCS. We conjecture the following:

- There are four equivalent {111} cleavage planes dispersed in the crystal coordinate. Any direction has a smaller angle than 54.74° against one of four <111> directions. The loading of any stress state provides relatively high normal stress on either of the {111} planes.
- In diamond-structured semiconductors including silicon, cleavage can be induced to occur on planes other than the primary cleavage plane^7^. For example, the surface energy of the {110} plane has been estimated to not be much larger than that of the {111} plane^7,45,46^. Our fracture surface observations in Figures 2a–d were consistent with this, where non-indexed, curved fracture surfaces were observed in addition to the {111} cleavage surface, especially in type Fx. Therefore, not only the {111} plane can be responsible for fracture.
- The existence of surface roughness can weaken the anisotropy effects by local stress fluctuation. This effect will be discussed in the next subsection.

From the foregoing discussion, we suggest that the first principal stress can be used as a sufficiently proper criterion for fatigue fracture. Note that the conclusion is not necessarily valid in cases where the stress state is much different, such as for unconcentrated uniform stress, as found in the tensile test specimen. In addition, the stress ratio of the cycle might be limited to *R*=−1 because the stress-ratio effect is not investigated in this report.

**Figure 5:**
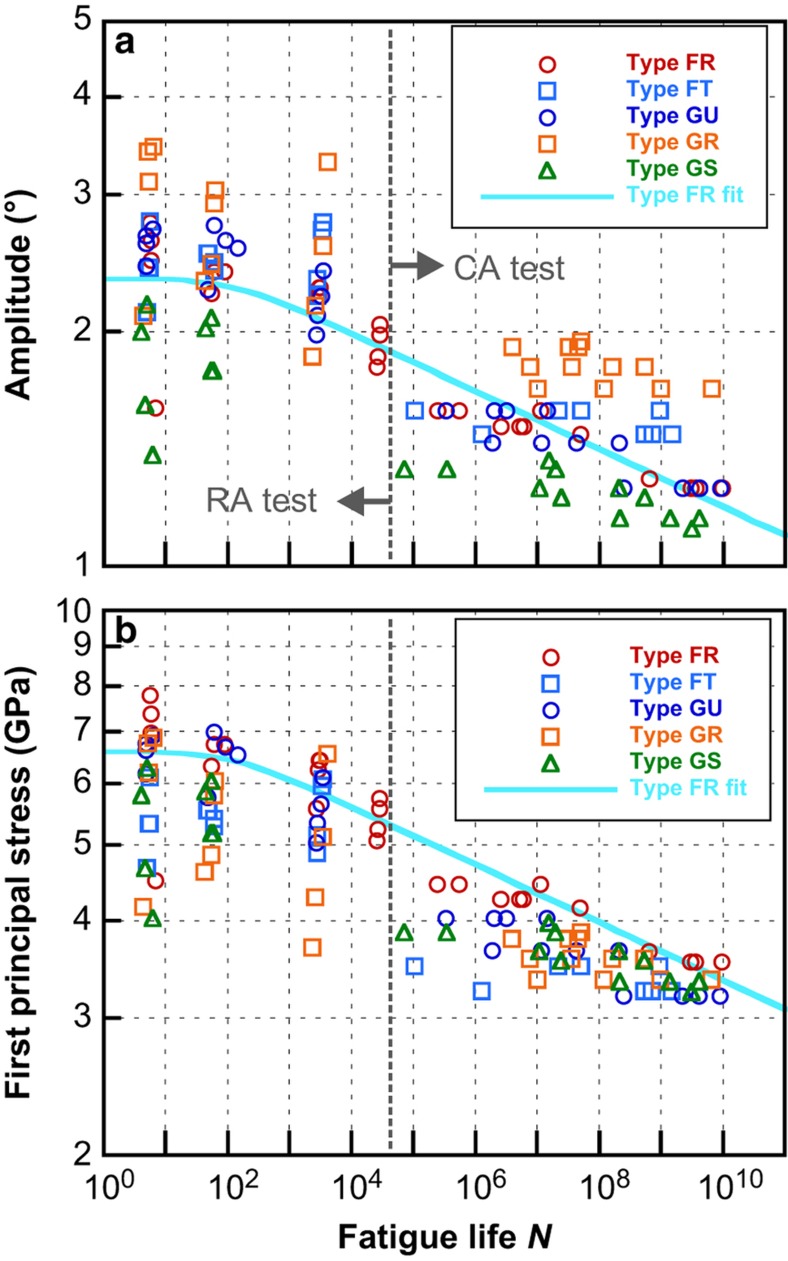
*S*–*N* plot of the fatigue test results for the five specimen types. Vertical axes are (**a**) deflection amplitude as original data and (**b**) normalized to the first principal stress using the calculated coefficient *k*. The solid curve shows the fitted line for type FR. The vertical dotted line indicates the boundary of the two test procedures.

We attempted normalization of the vertical axis of *S*–*N* curves to the first principal stress as shown in Figure 5. The original *S*–*N* data of the five types relative to the deflection amplitude (Figure 5a) seemed to gather around one fitted line after the normalization to the first principal stress (Figure 5b) using Equation (4), which demonstrated the validity of the first principal stress as a fatigue criterion. There were, however, many specimens that exhibited much lower strength than the fitted line. Most of them were specimens of types FT and GR in which a widely averaged stress distribution was expected, as shown in Figure 4i. We conjectured that this was a combination effect of surface imperfections, including the scallop structure and the stress distribution. If a single-point structural defect was assumed to exist on the notch surface, it would cause additional stress concentration and lower the strength^4,38^. The horizontal scallop structure itself has little effect on the stress state under an in-plane deformation. However, the scallop structure easily produces point-like defects combined with lithography or etching fluctuations, as seen in Figure 1d. The probability of the existence of such defects is considered to increase in the case of widely distributed stress as with types FT and GR.

These effects have been discussed as volume effects within the concept of Weibull statistics and investigated for micrometer-sized brittle materials in which the mean tensile strength becomes proportional to *V*_E_^−1/*m*^, where *V*_E_ is the effective volume and *m* is the Weibull modulus. The mean tensile strength decreases with increasing effective volume, even if the tensile strength and the Weibull modulus are unchanged^3^. The change in the mean tensile stress owing to anisotropic stress distribution has been estimated to be as much as 5% between SCS tensile test specimens with a similar semicircular notch (similar to types GU and GS)^9^. Corrections to the same extent are also expected in our experiments. The scattering of the *S*–*N* data of types FT and GR to the lower fatigue strength is considered a result of the volume effect owing to the wide stress distribution by elastic anisotropy. However, we could not include the volume effect quantitatively in this paper because the Weibull modulus was not obtained from the limited number of fatigue tests.

### Comparison with previous specimens

We have already compared the value of the crack growth exponent *n* in our current results with the previously tested specimens in the section 'Discussion on crack growth exponent *n'*. The previous types A and B had a design and crystal orientation identical to the new types FR and FT, respectively, excluding the fabrication process. The most distinct difference was the sidewall quality owing to the DRIE recipe optimization as described in the section 'Fabrication process and material'. We have measured^37^ that the strength factor between types A and B was 1.58, which was much larger than 1.12 between types FR and FT. This factor 1.58 was rather closer to the estimated strength factor 1.487 from the normal stress on the {111} plane (Table 2). This result suggested the possibility that the {111} normal stress could become a proper criterion in the case of extremely smooth surface specimens by excluding the averaging effect due to surface roughness instead of the first principal stress. Further tests will be required to confirm this hypothesis, although stable reproduction of such an ultra-smooth sidewall is a very difficult task. We simply note here that the quality of the etched surface can alter the orientation dependence and the crack growth exponent.

Surface modification processes have been attempted to improve the fracture properties of SCS structures by annealing in a hydrogen environment^47,48^ or by transient laser annealing^49^. However, careful comparison should be made because such annealing induces changes in material conditions such as the DRIE process alteration, not just in the surface morphology.

Finally, the initial crack length *a*_0_ is compared between the two fabrications. The normalized *S*–*N* plot (Figure 5b) reveals that *σ*_0_ without fatigue approximately coincided with 6.5 GPa. This value was almost the same as that in type A, 6.50 GPa, which corresponded to^17^ *a*_0_=7.9 nm using Equation (3). Similar values between two specimens with largely different surface roughness suggested that the initial crack length *a*_0_ was almost independent of the size of the surface scallops. The obtained values of *σ*_0_ can be considered static tensile strengths without fatigue. There is another report^50^ in which the static *σ*_0_ was independent of the scallop size. These observations suggest that the surface scallops formed by DRIE cannot be regarded directly as an initial crack for brittle fracture. Including other experimental results^48,51^ exhibiting the improvement effects of surface treatments, further detailed comparisons will be required. Because the strength evaluation methods (fracture or fatigue) give only phenomenological results, additional assistance from microscopic technologies will be strongly desired to further elucidate the surface condition effects.

## Conclusion

Repetitive bending fatigue tests were performed in five types of SCS specimens with different crystal orientations fabricated from {100} and {110} wafers. Fatigue lifetimes of 116 specimens in a wide range between 10^0^ and 10^10^ were obtained using amplitude-controlled oscillation methods. Fracture surface observation using SEM revealed that the {111} plane was the primary fracture plane. The crack propagation exponent *n* was estimated to be 27 and was found to be independent of the crystal orientation and dopant concentration. By contrast, the values were different between two fabrication lots using different processes. We conjectured that the value of *n* is governed strongly by the surface conditions of the etched sidewall. However, the fatigue strengths relative to the deflection angle were orientation dependent, and the factors were obtained to be 0.86 to 1.25 against the <110>-oriented specimens on {100}. The strength factors were compared with those obtained from FEM stress analyses. The calculated stress distributions showed strong orientation dependence, which was well-explained by elastic anisotropy. The comparison of the strength factors suggested that the first principal stress was a good criterion for fatigue fracture. Through comparisons with specimens tested in our previous report, additional discussions were provided on the tensile strength, initial crack length, volume effect, and effects of surface roughness such as scallops. We believe that these findings are helpful in improving the reliability of the design of MEMS devices.

## Figures and Tables

**Table 1:** Materials and typical properties of the five types of specimens along with two previously reported ones

Type	Plane	Direction	Dopant (Ω cm)	Typ. frequency (kHz)	Tested
FR	{100}	<110>	B (0.01–0.02)	42.9	28
FT	{100}	<100>	B (0.01–0.02)	40.8	20
GU	{110}	<110>	B (10–30)	43.5	24
GR	{110}	<100>	B (10–30)	39.4	24
GS	{110}	<111>	B (10–30)	45.2	20
A	{100}	<110>	B (0.1–0.5)	39.1	32
B	{100}	<100>	B (0.1–0.5)	36.1	16

**Table 2:** Fitted fatigue-strength factors against type FR relative to the deflection angle. Estimated strength factors *k*_FR_/*k* using FEM are also shown

Type	Observed strength factor	Estimated strength factor *k*_FR_/*k*		
		First principal	{111} normal	{111} shear
FR	1.00	1.000	1.000	1.000
FT	1.12	1.392	1.487	1.264
GU	1.00	1.106	0.862	0.894
GR	1.25	1.418	1.168	1.167
GS	0.86	0.970	0.721	0.972

Abbreviation: FEM, finite element method.

**Table 3:** Coefficients *k* between peak stress values (first principal, {111} normal, {111} shear) on the notch surface and deflection angle

Type	Coefficient *k* (GPa deg^−1^)		
	First principal	{111} normal	{111} shear
FR	2.816	2.045	1.090
FT	2.023	1.375	0.863
GU	2.546	2.372	1.219
GR	1.986	1.750	0.935
GS	2.902	2.838	1.121
